# Blind Deblurring Reconstruction Technique with Applications in PET Imaging

**DOI:** 10.1155/2009/718157

**Published:** 2009-06-11

**Authors:** Heng Li, Osama R. Mawlawi, Ronald X. Zhu, Yibin Zheng

**Affiliations:** ^1^Department of Radiation Physics, The University of Texas M. D. Anderson Cancer Center, Houston, TX 77030, USA; ^2^Department of Imaging Physics, The University of Texas M. D. Anderson Cancer Center, Houston, TX 77030, USA; ^3^GE Healthcare, Waukesha, WI 53188, USA

## Abstract

We developed an empirical PET model taking into account system blurring and a blind iterative reconstruction scheme that estimates both the actual image and the point spread function of the system. Reconstruction images of high quality can be acquired by using the proposed reconstruction technique for both synthetic and experimental data. In the synthetic data study, the algorithm reduces image blurring and preserves the edges without introducing extra artifacts. The localized measurement shows that the performance of the reconstruction image improved by up to 100%. In experimental data studies, the contrast and quality of reconstruction is substantially improved. The proposed method shows promise in tumor localization and quantification.

## 1. Introduction

In the past decade, positron emission tomography (PET) has rapidly become a popular diagnostic imaging modality for tumor detection and cancer staging. PET generates three-dimensional (3D) tomographic images of the distribution of positron-emitting radiotracers within an object, which provides quantitative functional information in vivo. However, the physics of the photon emission and detection process limits the resolution of PET images. The sensitivity and resolution of the PET system is a complex function of many factors, such as positron range, scattering, medium attenuation, photon noncollinearity, detector size, and intercrystal crosstalk. Iterative methods are often used in PET reconstruction because of the ability to handle the imaging system more precisely and to incorporate a prior information in the process.

**Figure 1:**
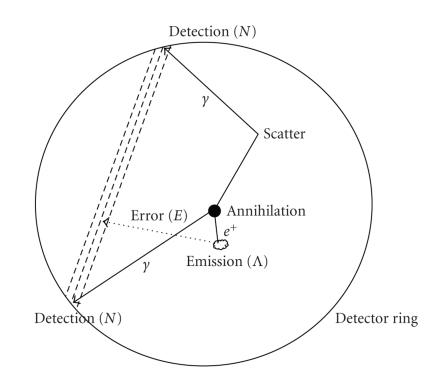
Diagram of PET data collection.

Figure 1  illustrates a PET imaging system [1]. A positron is emitted following a nuclear transmutation. After traveling a short distance (positron range), the positron annihilates with an electron and produces two photons of 0.511 MeV almost 180° to each other. The photons are then detected by the detector ring. Finally, by recognizing the coincidence photons, a line of response (LOR) can be established to determine the origin of the positron emission. It should be noted that LOR is not a real line due to the finite size of the detector elements. As shown in Figure 1, photons may be scattered to travel along a totally different direction upon reaching the detector ring, and the origin of the photon pair cannot easily be described by the LOR. Therefore, reconstructions obtained from iterative algorithms that determine the system matrix solely based on LOR or extended LOR are often blurry and contaminated by artifacts.

We recently developed a blind deblurring reconstruction technique to reduce blurring in pinhole SPECT imaging [2]. It is natural to apply a similar technique to PET imaging, because SPECT and PET share a similar underlying physic. Our approach is to model the discrepancy of LOR, or the measurement, and the true emission by an independent random variable *E*, as demonstrated in Figure 1, and to derive the maximum likelihood (ML) solution of the new system incorporating measurement error. In this report, we describe the development of the reconstruction algorithm.

## 2. Blind Deblurring Reconstruction

Equation (1) illustrates the widely used Expectation-Maximization (EM) algorithm for PET reconstruction [3]:

(1) $${\hat{\lambda }}_{i}^{n+1}=\frac{{\hat{\lambda }}_{i}^{n}}{\sum_{j}p_{ij}}\underset{j}{\sum }\frac{n_{j}p_{ij}}{\sum_{i^{\prime }}{\hat{\lambda }}_{i^{\prime }}^{n}p_{ij}},$$

where *p_ij_*, element (*i*, *j*) of system matrix *P*, denotes the probability of detecting an emission from voxel *i*, *i* = 1,…, *S*, at detector pair *j*, *j* = 1,…, *T*. The emitted photon pairs *Y*, radiotracer concentration Λ, and detected photon pairs *N* are as follows:

(2) $$\begin{array}{c} N=PY,\\ n_{j}=\underset{i}{\sum }\;p_{ij}y_{i},\\ y_{i}\sim \text{Poisson}\;\left(\lambda_{i}\right). \end{array}$$

The system matrix *P* can be factorized and correction factors can be applied as follows [4]:

(3) $$P=P_{\text{det}.\text{sens}}\;P_{\text{det}.\text{blur}\;}P_{\text{attn}}\;P_{\text{geom}}\;P_{\text{positron}},$$

where *P*_geom_ is the geometric projection matrix with each element (*i*, *j*) representing the probability of a photon pair produced in voxel *i* reaching detector pair *j*, ignoring attenuation, and assuming perfect photon-pair collinearity. The values in the matrix are defined by the geometries of each LOR. *P*_attn_ and *P*_det.sens_ are the attenuation correction and detector sensitivity normalization factors, respectively. *P*_positron_, the positron range factor, is usually omitted in ^18^F studies in which the positron range is submillimeter, and *P*_det.blur_ is the detector blurring factor used to model photon-pair noncollinearity, intercrystal scatter, and penetration, which cause the same photon detected by several adjusting detector elements. In general, the correction can be viewed as weighting and broadening the LOR on top of *P*_geom_.

In this work, instead of adding correction factors to the system matrix *P*, we model the uncertainty caused by positron range, photon emission angle, scatter, and detector response as a blurring factor *E* that introduces measurement error, which is a random variable independent from emission *Y*, and modify the system model accordingly. The measurement error introduces blur in the reconstruction image, and a blurred PET reconstruction can be viewed as the convolution of a low-pass point spread function (PSF) with the actual image, where both the PSF and the actual image are unknown in practice. Inspired by the blind deconvolution algorithm introduced by Holmes [5] and along the same line of research we have applied to SPECT imaging [2], we formulated a blind deblurring reconstruction algorithm. The algorithm is a modified EM algorithm that includes a convolution kernel to model the blurring factor *E*. The algorithm consists of two iterative updates, instead of one, to reconstruct both the object and the PSF.

 In a PET imaging system, suppose *s*_*k*_, *k* = 1 ⋯ Λ, *s*_*k*_ ∈ {1 ⋯ *S*}, where Λ is the total number of emitted photons, denotes the index of location from which the *k*th pair of photons are emitted, and *t*_*k*_, *t*_*k*_ ∈ {1 ⋯ *T*} denotes the location where the *k*th pair of photons are detected, as shown in Figure 1. We call these emission locations “true emission points.” A finite number of these points form an inhomogeneous Poisson random-point process having the intensity function *λ*_*i*_. With the presence of measurement error, the positional measurement of each emission point is corrupted by a random translation. Let *e*_*k*_, *e*_*k*_ ∈ {1 ⋯ *S*} denote this error vector, then the measured data for detector pixel *j* is related to *s*_*k*_ and *e*_*k*_ by

(4) $$\begin{array}{c} P\left(t_{k}=j\;|\;s_{k}+e_{k}=i\right)=p_{ij},\\ n_{j}=\overset{\Lambda }{\underset{k=1}{\sum }}p_{(s_{k}+e_{k})j}. \end{array}$$

Here, *e*_*k*_ is statistically independent of all *s*_*k*_'s, and they are assumed to be all statistically independent of each other for all photons emitted and identically distributed with a probability density *g*_*i*_, which is also the PSF of the PET system. In addition, the set of error vectors **E** = {*e*_1_ ⋯ *e*_Λ_} also constitutes an inhomogeneous Poisson random point process with intensity function *γ*_*i*_ = Λ*g*_*i*_ [6]. 

Now let *y*_*i*_ be the actual number of photon pairs emitted from voxel *i*, and let *b*_*i*_ be the corresponding error vectors within voxel *i*; they then follow the Poisson distribution with mean *λ*_*i*_ and *γ*_*i*_, respectively, from the results above. We then use the EM algorithm to find the maximum likelihood solution of the system. In our application, *n*_*j*_ and Λ are known measured data, whereas *y*_*i*_ and *b*_*i*_ are unknown data. Here we note the set of true emission vectors **Y** and the set of error vectors **B** as

(5) $$\begin{array}{c} \text{Y}=\left\{y_{1},y_{2},y_{3},\ldots \right\},\\ B=\left\{b_{1},b_{2},b_{3},\ldots \right\}. \end{array}$$

Then the log likelihood of **I** can be expressed as

(6) $$L\left(\text{I}\;|\;\lambda \right)=-\underset{i}{\sum }\lambda_{i}+\underset{i}{\sum }\text{ln}\left(\lambda_{i}\right)y_{i},$$

where ***λ*** is the vector notation for all *λ*_*i*_. Similarly, the log likelihood of **B** can be written as

(7) $$L\left(\text{Y}\;|\;g,\Lambda \right)=-\underset{i}{\sum }\gamma_{i}+\underset{i}{\sum }\text{ln}\left(\gamma_{i}\right)b_{i},$$

where **g** is the vector notation for all *g*_*i*_. The log likelihood of the complete data then can be equivalently expressed in two ways, assuming **Y** or **B** being known, that is,

(8) $$\begin{array}{ll} L\left(I,\text{Y}\;|\;\lambda ,g,\Lambda \right) & =L\left(I\;|\;\lambda ,g,\Lambda \right)+l\left(\text{Y}\;|\;\lambda ,g,\Lambda \right)\\ & =-\underset{i}{\sum }\;\lambda_{i}+\underset{i}{\sum }\;\text{ln}\left(\lambda_{i}\right)y_{i}+\overset{\Lambda }{\underset{k=1}{\sum }}\;\text{ln}\left(g\left(s_{k}\right)\right)\\ & =-\underset{i}{\sum }\;\gamma_{i}+\underset{i}{\sum }\;\text{ln}\left(\gamma_{i}\right)b_{i}+\overset{\Lambda }{\underset{k=1}{\sum }}\;\text{ln}\left(\frac{\lambda \left(e_{k}\right)}{\Lambda }\right). \end{array}$$

By maximizing (8) using a derivation similar to those of Holmes [5] and Li [2], the following iteration can be shown to converge to the maximum likelihood estimate of *λ*_*i*_ and *g*_*i*_:

(9) $${\hat{\lambda }}_{i}^{n+1}={\hat{\lambda }}_{i}^{n}\underset{j}{\sum }\frac{n_{j}\sum_{k}g_{i-i^{\prime \prime }}p_{i^{\prime \prime }j}^{}}{\sum_{i^{\prime }}\left({\hat{\lambda }}_{i^{\prime }}^{n}*g_{i\prime }\right)p_{i^{\prime }j}^{}},$$

(10) $${\hat{g}}_{i}^{n+1}=\frac{{\hat{g}}_{i}^{n}}{\Lambda }\underset{j}{\sum }\frac{n_{j}\sum_{i^{\prime \prime }}\lambda_{i-i^{\prime \prime }}p_{i^{\prime \prime }j}^{}}{\sum_{i^{\prime }}\left(\lambda_{i^{\prime }}^{}*{\hat{g}}_{i^{\prime }}^{n}\right)p_{i^{\prime \prime }j}^{}},$$

where ∗ denotes convolution. The initial *λ*_*i*_^0^ is an image of all 1's, and *g*_*i*_^0^ is the same image normalized to 1. Equations (9) and (10) are then evaluated to acquire a new pair of estimates of *λ* and *g*. The PSF of the system is assumed to be real, nonnegative, band limited, and limited in extend. Letting *F*_*z*_ be the frequency components of the PSF that are known to be zero and *F*_*r*_ be the space components of the PSF that are known to be zero, the band-limited and limited-extend constraints are incorporated by executing the following steps in each iteration.

1. The Fourier transform of ${\hat{g}}^{n+1}$ is taken, and any frequency components that lie within *F*_*z*_ are set to zero.
2. The inverse Fourier transform in step 1 above is taken, and any negative or complex values or values within *F*_*r*_ in the spatial domain are set to zero.

The first step of the process ensures the band-limited constraint, and the second step ensures the reality, nonnegativity, and limited-extend of the PSF. Realness and nonnegativity are implicitly applied to *λ*. Equations (9) and (10) and steps (1) and (2) are then iterated until convergence occurs.

The blind deblurring reconstruction algorithm estimates both the spatial radioactivity distribution and the system PSF from the set of blurred projection images. The iteration for reconstruction can be understood as replacing the forward projector in the original EM (denominator of (9)) with the new projector using the convolved radioactivity map, and the iteration for solving the PSF can be understood as blind deblurring. This iteration differs from the general image-blind deconvolution in the sense that the kernel is partly known: *p*_*ij*_, the system matrix, is in fact part of the blurring kernel. The more precise the model *p*_*ij*_ is, the closer the remainder of blurring kernel, or *g*, is to a true delta function. In addition, instead of deconvolving an image where both the input and output are 2D images, the input of blind deblurring reconstruction is a series of projection images, and the output is a 3D-image array. This property gives us much more knowledge of the noise distribution within the object, because instead of a single-shot image, we now essentially have multiple samples for each point in the 3D array (although mixed with other points). Both simulation and experimental data were used to validate and evaluate the performance of the blind deblurring EM (BDEM) technique.

## 3. Methods

### 3.1. Monte Carlo Simulation

PET simulations were performed utilizing the NCAT phantom covering the chest region [7, 8]. The PET emission data were generated using the Monte Carlo method, with 1 million counts per slice using a geometry corresponding to the design of the GE discovery DSTE PET/CT scanner (GE Healthcare, Milwaukee, WI, USA). The images were reconstructed using the standard EM and the BDEM. The reconstruction image sets were then evaluated using visual inspections and line profiles. The EM reconstruction image was postsmoothed using a Gaussian kernel of 1 pixel full width at half medium, and no post processing was done for BDEM.

### 3.2. Convergence Study

One major concern with regard to the type of blind deblurring technique used is the effectiveness and convergence of the algorithm. Two hundred iterations of EM and BDEM reconstructions of the NCAT phantom were performed to evaluate the convergence property of the BDEM algorithm. In each iteration, the measurement log-likelihood *L* of the reconstructed image *λ* for EM was calculated as follows [6]:

(11) $$L\left(\lambda \;|\;N\right)=\overset{}{\underset{j}{\sum }}(n_{j}\text{log}\underset{i}{\sum }\;p_{ij}\lambda_{i}-\underset{i}{\sum }\;p_{ij}\lambda_{i}-\text{log}\;n_{j}!)$$

whereas for BDEM, the log-likelihood *L* of the reconstruction image was calculated as

(12) $$L\left(\lambda \;|\;N\right)=\overset{}{\underset{j}{\sum }}(n_{j}\text{log}\underset{i}{\sum }\;p_{ij}\left(\lambda_{i}*g_{i}\right)-\underset{i}{\sum }\;p_{ij}\left(\lambda_{i}*g_{i}\right)-\text{log}\;n_{j}!).$$

Two different settings of limited-extend constraint were used for BDEM reconstruction, and the difference in log-likelihood was compared.

### 3.3. Comparison with Image Deconvolution

The blurred reconstruction image could always be modeled as a convolution of the true radioactivity with a blurring kernel *h*_*i*_:

(13) $$f_{i}=\lambda_{i}*h_{i},$$

where *f*_*i*_ is the standard EM reconstruction image and ∗ denotes 2D linear convolution. Assuming *h*_*i*_ can be estimated, one could first compute *f*_*i*_ using standard EM iterations (with ${\hat{\lambda }}_{i}$ being replaced by ${\hat{f}}_{i}$) and then deconvolve ${\hat{f}}_{i}$ with *h*_*i*_. However, the ${\hat{\lambda }}_{i}$ so obtained is not the maximum likelihood estimate of *λ*_*i*_ given the measurement error; also, the kernel *h*_*i*_ is generally a complex unknown function and is hard to measure. We used the Wiener filter and image blind deconvolution algorithm to denoise and deconvolve the EM reconstruction image, and compared the results with the BDEM reconstruction.

### 3.4. Physical Phantom Study

A physical phantom study with a water-tank phantom containing 4 spheres of diameters 1.5 cm, 2.0 cm, 3.0 cm, and 3.0 cm was conducted to assess the performance of a BDEM algorithm for a real scanner. Both the water tank and the spheres were filled with ^18^FDG, with activity concentrations of 0.20 *μ*Ci/cc and 1.01 0.20 *μ*Ci/cc, respectively. The tank was imaged with a GE Discovery DSTE PET/CT scanner (GE Healthcare) in 2D list-mode; the sinogram was extracted and reconstructed using the EM and BDEM techniques.

## 4. Results and Discussion

### 4.1. Monte Carlo Simulation

**Figure 2:**
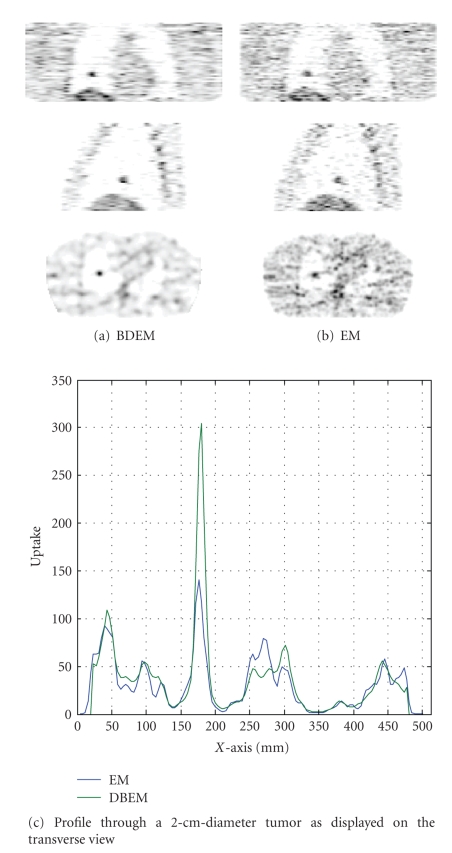
Reconstruction of NCAT utilizing EM and BDEM algorithms. From top to bottom are the coronal, sagittal, and transverse views.

Figure 2  compares the image slices through a 2-cm-diameter tumor in the right lung produced by the EM and BDEM reconstruction techniques. It is clear that the image quality of the BDEM reconstruction is superior to that of the EM reconstruction. The contrast of the lesion of interest is greatly improved, edges are preserved, and artifacts are suppressed. The transverse-view line profile across the tumor also confirms the improvement. The peak contrast of the tumor in the BDEM reconstruction is double that of the EM reconstruction. Although no postsmoothing was performed for the BDEM reconstruction, the noise level in the background of the BDEM reconstruction is lower than that of the EM reconstruction, with postsmoothing by a 1-pixel full width at half medium Gaussian kernel. The mean and standard deviation of a small region of interest in the background region are 36.20 and 25.12 (resp.) for the Gaussian-smoothed EM and 34.83 and 21.28 (resp.) for BDEM, with no postprocessing.

### 4.2. Convergence Study

Figure 2  shows the log-likelihood as a function of iteration numbers as calculated in (11) and (12). The measurement log-likelihood of BDEM is a monotonic increasing function of iteration number, which confirms the convergence of the algorithm. It also varies with different PSF constraints and lies within the envelope defined by the EM algorithm, as demonstrated in the figure. Therefore, setting a proper constraint on PSF is important. If the initial PSF is a delta function and no other constraint is set, the BDEM would regress to the regular EM solution. Figure 2  also shows the log-likelihood of BDEM with a 10 × 10- and a 20 × 20-PSF spatial constraint being applied for a 128 × 128 reconstruction. The small discrepancy in the convergence of two different constraints indicates that BDEM is not a strong function of PSF constraint: as long as the constraint is reasonable, BDEM would converge to very similar solutions. The figure also indicates that BDEM converges a little faster than conventional EM in terms of number of iterations. It should be noted, however, that the amount of computation for each BDEM iteration is about twice that of a computation of an EM iteration. We have not tested this premise, because the algorithm converges reasonable fast even without order-subset, but the principle of order-subset EM (OSEM) should be applied if necessary.

### 4.3. Comparison with Image Deconvolution

**Figure 4:**
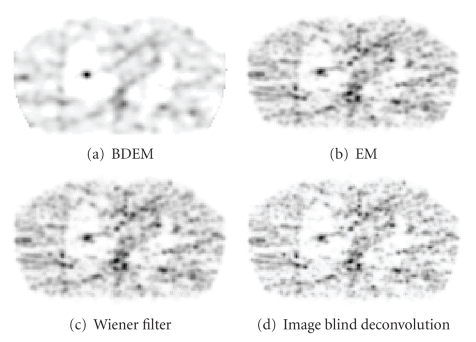
EM and BDEM compared to image deblurring techniques.

The results obtained by deblurring the EM reconstruction image with Wiener filter and the image-blind deblurring technique were displayed and compared with the results of the EM and BDEM reconstructions (Figure 4). These image deconvolution results are clearly inferior compared to BDEM reconstruction. The images are still noisy, and the visual improvement of deconvolution is limited. Our explanation is that the BDEM algorithm utilizes the statistical information of both emission and measurement noise in the projection data, which is loss in the reconstruction image. The noise in the projection data commonly appears as streaks or other local or global artifacts in the reconstruction image, which makes it much harder to either identify or clear just from the reconstruction image.

### 4.4. Physical Phantom Study

Figure 4  shows one slice of water phantom with spheres. As with the Monte Carlo study, the image contrast of the BDEM has been improved over the conventional EM, and the mass and edges are well preserved in the reconstruction. The image blurring is reduced, and the contrast is greatly improved (up to 50%), as observed from the line profile from the transverse slice. The shape of the lesions is not distorted, indicating that the algorithm preserves the general shape of objects.

## 5. Conclusion

In this work, we demonstrated highly desired reconstruction results with no complex assumption about the imaging system or the object. The blind deblurring reconstruction technique can significantly improve the quality and contrast of the reconstruction as demonstrated in both simulation and experimental scans. This algorithm does not only reconstruct the radiotracer map, but also determines the PSF of the system. The masses and edges are well preserved in the reconstruction image, which can be extremely useful when doctors need to localize, segment, or tally the activities in the possible tumor. Future studies would involve the application of this system in patient imaging and quantitative studies.

## Figures and Tables

**Figure 3:**
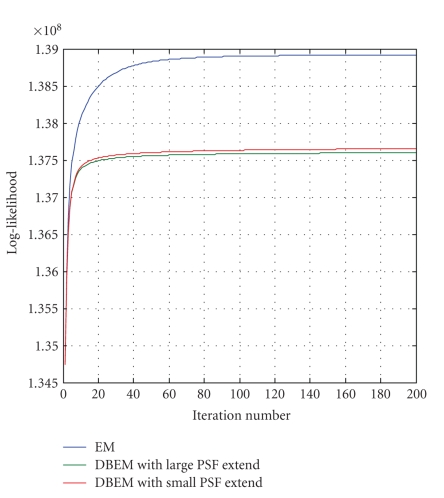
Log-likelihood of EM and BDEM algorithms as a function of iteration number. The two BDEM plots have different PSF extend constraints; small PSF extend limits the PSF to 10 × 10, whereas large extend limits the PSF to 20 × 20. The image size is 128 × 128 pixels.

**Figure 5:**
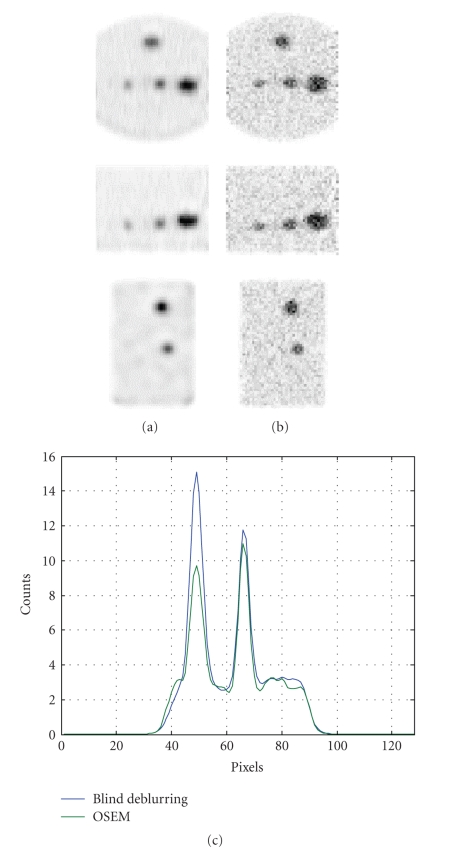
Water phantom reconstruction. From top to bottom are the coronal, sagittal, and transverse views.

## References

[1] Bailey DL, Townsend DW, Valk PE, Maisey MN (2004). *Positron Emission Tomography, Basic Sciences*.

[2] Li H, Qiao F, Mawlawi OR, Zheng Y, Zhu RX Blind deblurring reconstruction technique with applications in PET imaging.

[3] Shepp LA, Vardi Y (1982). Maximum likelihood reconstruction for emission tomography. *IEEE Transactions on Medical Imaging*.

[4] Qi J, Leahy RM, Cherry SR, Chatziioannou A, Farquhar TH (1998). High-resolution 3D Bayesian image reconstruction using the microPET small-animal scanner. *Physics in Medicine and Biology*.

[5] Holmes TJ (1992). Blind deconvolution of quantum-limited incoherent imagery: maximum-likelihood approach. *Journal of the Optical Society of America. A*.

[6] Ollinger JM, Fessler JA (1997). Positron-emission tomography. *IEEE Signal Processing Magazine*.

[7] Segars WP (2001). *Development and application of the new dynamic NURBS-based cardiac-torso (NCAT) phantom*.

[8] Qiao F, Pan T, Clark JW, Mawlawi OR (2007). Region of interest motion compensation for PET image reconstruction. *Physics in Medicine and Biology*.

